# Characterization of chitin and chitosan derived from *Hermetia illucens*, a further step in a circular economy process

**DOI:** 10.1038/s41598-022-10423-5

**Published:** 2022-04-22

**Authors:** Micaela Triunfo, Elena Tafi, Anna Guarnieri, Rosanna Salvia, Carmen Scieuzo, Thomas Hahn, Susanne Zibek, Alessandro Gagliardini, Luca Panariello, Maria Beatrice Coltelli, Angela De Bonis, Patrizia Falabella

**Affiliations:** 1 Department of Sciences, University of Basilicata, Potenza, Italy; 2 Spinoff XFLIES s.r.l, University of Basilicata, Potenza, Italy; 3 Fraunhofer Institute for Interfacial Engineering and Biotechnology IGB, Stuttgart, Germany; 4 R & D Texol, Alanno, 65020 Pescara, Italy; 5 Department of Civil and Industrial Engineering, University of Pisa, Pisa, Italy

**Keywords:** Biotechnology, Bioinspired materials

## Abstract

Due to their properties and applications, the growing demand for chitin and chitosan has stimulated the market to find more sustainable alternatives to the current commercial source (crustaceans). Bioconverter insects, such as *Hermetia illucens*, are the appropriate candidates, as chitin is a side stream of insect farms for feed applications. This is the first report on production and characterization of chitin and chitosan from different biomasses derived from *H. illucens*, valorizing the overproduced larvae in feed applications, the pupal exuviae and the dead adults. Pupal exuviae are the best biomass, both for chitin and chitosan yields and for their abundance and easy supply from insect farms. Fourier-transform infrared spectroscopy, X-ray diffraction and scanning electron microscope analysis revealed the similarity of insect-derived polymers to commercial ones in terms of purity and structural morphology, and therefore their suitability for industrial and biomedical applications. Its fibrillary nature makes *H. illucens* chitin suitable for producing fibrous manufacts after conversion to chitin nanofibrils, particularly adults-derived chitin, because of its high crystallinity. A great versatility emerged from the evaluation of the physicochemical properties of chitosan obtained from *H. illucens*, which presented a lower viscosity-average molecular weight and a high deacetylation degree, fostering its putative antimicrobial properties.

## Introduction

Chitin is a natural biopolymer, widely spread on Earth secondary only to cellulose^1^. It is a linear polymer of *N*-acetyl-d-glucosamine (GlcNAc), structurally similar to cellulose, from which it differs for acetamido groups at C_2_ position of the glucose unit^2–4^. In nature three different crystalline forms of chitin, α-, β- and γ, have been found^2^. α-chitin is the most common form, with strong intermolecular and intramolecular bonds^5^ and an anti-parallel orientation of its chains. It is present in yeasts, fungi, crustaceans, insects and sponges^6,7^. β-chitin is a constituent of the *Loligo* squid’s pen, pogonophoran tubes and diatoms’ spines^8^ and it has a parallel arrangement of chains, resulting in lower degree of crystallinity than the α-form^2,9^. γ-chitin, a mixture of α- and β-form, is found in the stomach of *Loligo* and in the cocoon fibers of the *Ptinus* beetle^2^. Chitin, for its nature, is not soluble in most organic solvents and water and this makes it difficult to use^3,4^. However, through a deacetylation process, chitin is converted into chitosan, which is soluble in acidic solutions^10,11^. Chitosan is a copolymer consisting of glucosamine and N-acetylglucosamine^12,13^. The amino groups, responsible for the basic behavior and cationic properties, are crucial for polymer reactivity^14^. Degree of deacetylation, molecular weight, crystallinity and viscosity are some of the main parameters that affect the chemical and physical properties, and final activity of chitosan^15,16^.

Biodegradability, biocompatibility, non-toxicity, haemostaticity, bioadhesiveness and immunostimulant activity are some useful properties of chitin and chitosan that make them polymers of economic interest^5,10,17–19^. Chitin and chitosan find application in the food industry, agriculture, wastewater treatment, tissue engineering, biomedical, biotechnological, sanitary and cosmetic sectors, and in the textile and paper industries^12,20–29^.

Currently, the largest amount of chitin and chitosan comes from waste of the fishing industry, that has a chitin content of 15–40%^30–33^. Annually, the industrial production of chitin from fishery waste amounts to around 1 × 10^11^ tons^34^. Based on the compound annual growth rate of 15.4% occurred from 2016 to 2021, the global chitin market is estimated to exceed 155 thousand tons by the year 2022^35^. However, this source has some limitations related to its seasonal availability, location mainly on coastal areas, and consequent transport costs and emissions to be borne to supply other areas^36,37^. In addition to crustaceans, this polymer is also structurally present in the cell wall of fungi and, especially, in the exoskeleton of insects^3,4,33,38,39^. Farmed insects have the potential to be a suitable and more sustainable alternative source of chitin and chitosan, being not subjected to seasonality, easily available, adaptable and resistant to a wide range of pathogens^40,41^. Furthermore, insect farms, carried out for the production of animal feed and waste management, are developing worldwide^42^. These farms generate side streams consisting of chitin-rich biomasses that could be subsequently processed for the extraction of this polymer. Among insects, *Hermetia illucens*, the black soldier fly, is reared by about 80% of the European insect farms thanks to its excellent bioconversion ability^43–45^: larvae are able to feed on decaying organic substrates transforming them into a biomass of high biological value, composed of protein and lipid to use mainly in feed^46,47^, but also in energetic and cosmetic fields^48,49^, or to extract molecules of pharmacological interest^50–53^. *H. illucens* life cycle consists of five larval stages, followed by pre-pupal and pupal stages from which the adult fly emerges. Chitin can be extracted from each stage^54,55^. *H. illucens* breeding thus allows for a continuous supply of pupal exuviae, the exoskeleton part that is released after moult, and dead adults, both of which are chitin-rich waste materials^56^. Hence, the breeding yield strategies could be improved by recovering this high-quality biopolymer from these byproducts^57^.

The present work aims to characterize the process of chitin extraction and chitosan production from larvae, pupal exuviae and dead adults of *H. illucens*. Specifically, the objectives are: (i) isolation of chitin and production of chitosan from the three different *H. illucens* biomasses (larvae, pupal exuviae and dead adults); (ii) assessment of the effectivity of the chitin purification process; (iii) evaluation of the physicochemical properties of the obtained chitosan; (iv) characterization of both polymers by FTIR, XRD and SEM; and (v) comparison between the characteristics of the samples obtained from *H. illucens* and those of the commercially available polymers.

## Results and discussion

### Composition of raw samples

Results regarding composition of larvae, pupal exuviae and dead adults of *H. illucens* related to the dry mass of the samples are reported in Table 1.

**Table 1:** Composition of raw *H. illucens* larvae, pupal exuviae and dead adults used for chitin extraction and chitosan production.

	Larvae	Pupal exuviae	Dead adults
Dry mass %	22.0 ± 0.8^b^	94.0 ± 0.7^a^	93.0 ± 0.9^a^
Minerals %	12.5 ± 0.1^b^	16.0 ± 0.2^a^	8.1 ± 0.5^c^
Proteins %	38.7 ± 1.6^b^	30.0 ± 2.8^c^	49.0 ± 0.4^a^
ADF %	22.0 ± 3.7^b^	53.5 ± 1.8^a^	23.7 ± 2.4^b^
ADL %	9.5 ± 2.2^b^	28.0 ± 1.2^a^	10.8 ± 2.4^b^
Chitin % (ADF − ADL)	12.4 ± 1.7^b^	25.5 ± 0.5^a^	12.8 ± 1^b^
Lipids %	23.0 ± 0.3^a^	5.0 ± 0.1^c^	19.7 ± 0.9^b^
Others %	13.4 ± 0.8	23.5 ± 1.3	10.4 ± 0.3

Additional components defined as "others" have been calculated by the difference between 100 and the percentage of the other elements, experimentally determined. Data are expressed as mean ± standard deviation. Different letters in a row indicate significant differences among the different samples in the percentage of each component (p < 0.05) (data analysed with one-way ANOVA and Tuckey post-hoc test).

*ADF* acid detergent fibre, *ADL* acid detergent lignin.

The three samples had a significantly different composition. Larvae had the greatest amounts of lipids (23.0%), adults the highest protein content (49.0%), while minerals (16.0%) and fibres (53.3%) were higher in pupal exuviae. Pupal exuviae were also the insect sample with the highest chitin content (25.5%), representing the biomass of choice for the production of chitin and its derivative, chitosan. According to literature, whole insects contain 30–60% protein, 10–25% lipid, 5–25% chitin and 2–10% minerals^31,58–61^. All these components are reported in wide ranges, as the composition of insects varies, especially depending on their species and stage of development. Our data regarding larvae, pupal exuviae and dead adults from the *H. illucens* are within these ranges. Similarities were also found in the average composition of the different developmental stages of *H. illucens* reported by other authors^62–65^, except for lipids. Indeed, our samples had a lower lipid content than those reported for the same insect biomass (3–40%, 8 and 27% for larvae, exuviae and adults, respectively)^62,63^. These differences could be due to the different feeding substrate of larvae and to the different methodology used for the analysis of lipid content.

### Chitin purification from *H. illucens*

**Figure 1:**
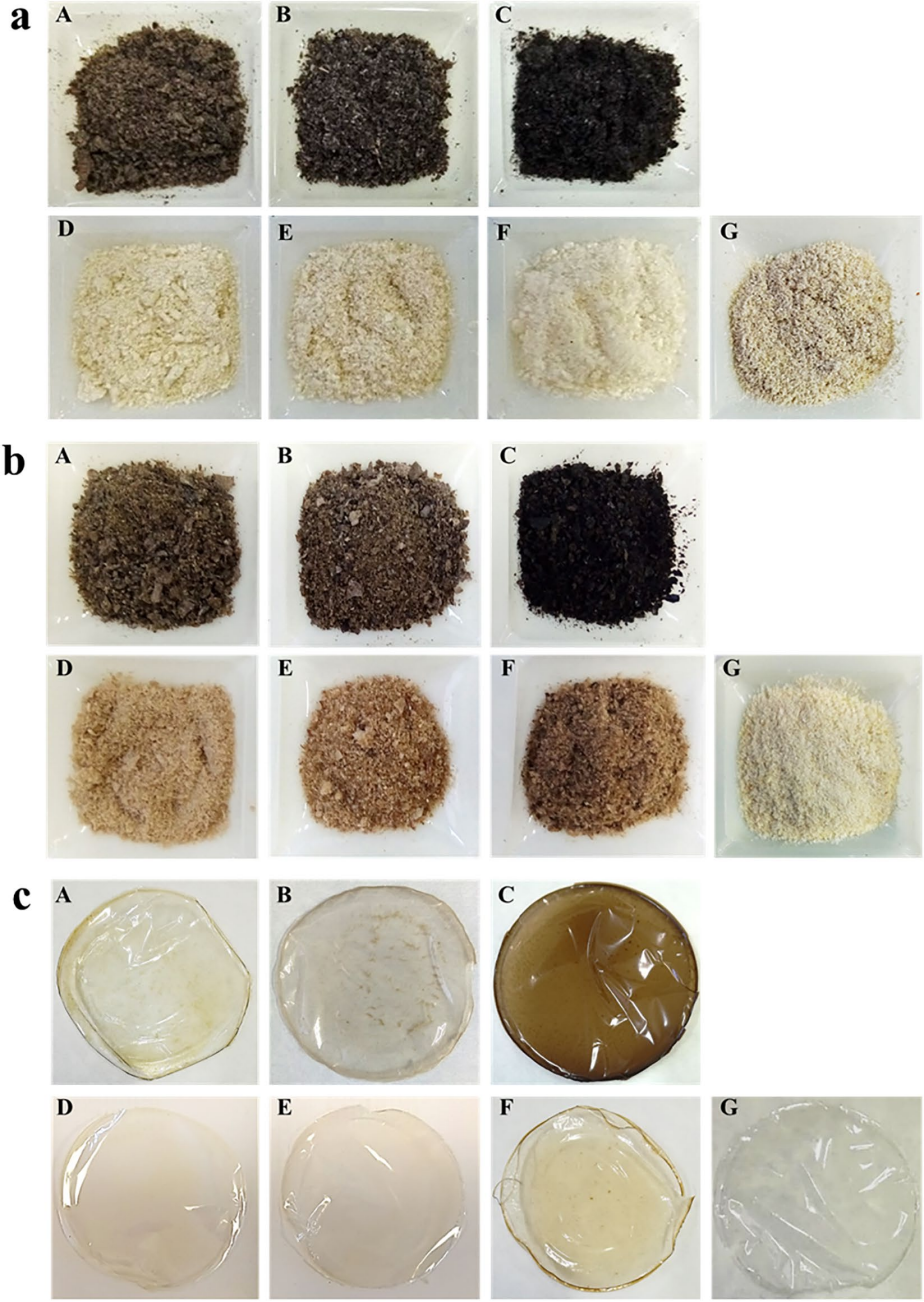
**(a)** Unbleached (A,B,C) and bleached (D,E,F) chitins extracted from *H. illucens* larvae (A,D), pupal exuviae (B,E) and dead adults (C,F). Commercial chitin produced from crustaceans is also shown (G); **(b)** unbleached (A,B,C) and bleached (D,E,F) chitosan produced from *H. illucens* larvae (A,D), pupal exuviae (B,E) and dead adults (C,F). Commercial chitosan derived from shrimp shells is also shown (G); **(c)** films obtained from unbleached (A,B,C) and bleached (D,E,F) chitosan produced from *H. illucens* larvae (A,D), pupal exuviae (B,E) and dead adults (C,F). Film obtained from commercial chitosan is also reported (G).

Chitin was purified from *H. illucens* larvae, pupal exuviae and dead adults (Fig. 1a), leaving a part of each chitin sample as it was, unbleached (Fig. 1a(A–B–C)), and another bleached (Fig. 1a(D–E–F)). Bleaching treatment resulted in a chitin with a whiteness similar to commercial chitin and, even brighter, in case of adults bleached chitin (Fig. 1a(F)). A qualitative assessment of the insect chitin purification process is generally reported in the literature, instead, data providing a comprehensive quantitative assessment is lacking. Even less data is available on the extraction process of chitin from *H. illucens*. Results of the chitin extraction process were expressed in terms of efficiency of the purification method applied, chitin recovery during it, purity and yield of the obtained chitin.

#### Biomass recovery and chitin yield

Biomass recovery from the insect samples after each purification step was calculated for all samples (Table 2). Due to very few numerical data providing this value, our results can only be compared with values reported by Khayrova et al*.*^57,65^.

**Table 2:** Biomass recovery (%) from larvae, pupal exuviae and dead adults after demineralization (DM/RAW) and deproteinization (DP/DM), efficiency (%) of DM and DP, and yields (%) of unbleached and bleached chitin related to the original raw insect biomass.

	Larvae	Pupal exuviae	Adults
DM/RAW %	66 ± 1^b^	77 ± 2.7^a^	68 ± 1.1^b^
DM efficiency %	82.0 ± 0.1^b^	85.0 ± 1.5^a^	87 ± 0.4^a^
DP/DM %	19 ± 1.2^b^	41 ± 0.8^a^	14 ± 0.8^c^
DP efficiency %	94.0 ± 0.2^b^	92.0 ± 1.2^b^	97.0 ± 1^a^
Yield of unbleached chitin %	13 ± 0.7^b^	31 ± 1.6^a^	9 ± 0.4^c^
Yield of bleached chitin %	10 ± 0.7^b^	23 ± 1.9^a^	6 ± 0.1^c^

Data are expressed as mean ± standard deviation*.* Different letters in a row indicate significant differences in the percentage of chitin recovery, DM and DP efficiency and yield among the different insect samples (*p* < 0.05) (data analyzed with one-way ANOVA and Tuckey *post-hoc* test).

The highest recovery was obtained with pupal exuviae during all extraction procedures, on the other hand adults had the lowest. Specifically, biomass recovery after demineralization (DM) of both larvae and pupal exuviae was similar to or slightly higher than that obtained by Khayrova et al*.*^57,65^ (58 and 74%, respectively), in contrast to that from adults which was lower (68% vs 87%)^65^. After deproteinization (DP), lower amounts were recovered from demineralized chitin than values reported by Khayrova et al*.*^57,65^ (46, 77 and 24% for larvae, exuviae and adults, respectively). Values reported by Hahn et al*.*^66^, referring to larval exoskeletons of *H. illucens* (79% after DM and 34% after DP) were close to those for pupal exuviae. These results revealed correlations between protein content of the starting sample and the chitin recovery after the DP step. Indeed, the adults, the sample with the highest starting protein content (49%), had the lowest percentage of chitin recovery after DP (14%); in contrast, pupal exuviae, with the lowest starting protein (30%), gave the highest percentage of recovery (41%). In accordance with the composition of raw samples of *H. illucens* (Table 1), adults contained, similarly to larvae, a higher protein and lipid content (about 70%) than that reported for pupal exuviae (about 35%), resulting in a lower final chitin content. These compositional differences can be explained considering that larvae and adults catabolize lipids and proteins to produce the energy necessary for growth and reproduction, respectively; pupal exuviae on the other hand, represent a waste product resulting from the moulting process and are mainly composed of chitin.

Yields of unbleached chitin from larvae, pupal exuviae, and adults were 13, 31, and 9%, respectively (Table 2). After bleaching, these yields decreased slightly to 10, 23, and 6%, but no significant differences were found between the two values for each sample (larvae *Χ*^2^ = 0.2, *p* = 0.66; pupal exuviae *Χ*^2^ = 1.24, *p* = 0.26; adults *Χ*^2^ = 0.29, *p* = 0.59), thus demonstrating the bleaching treatment did not affect chitin yield but favored its degree of purity. Chitin yields are within or higher than the average range reported for chitin from insects (5–15%)^32^ and chitin from crustaceans (5–30%)^20,67–69^. For insects, the highest values, between 31 and 36%, were obtained from larval exoskeletons of *H. illucens*^66^, cicada sloughs^70^ and adults of *Apis mellifera*^71^. The yields of bleached chitin, obtained in the present work, were comparable to or higher than those obtained by other authors using the same developmental stage of *H. illucens*^63,64,72–74^. In a similar range (6–26%) are the yield values obtained by Zhou et al*.*^75^ using natural deep eutectic solvents for the purification of chitin from *H. illucens* prepupae. It should be considered that the yield of chitin can vary greatly depending on the species, the developmental stages, the body parts, and the extraction methods used.

#### Efficiency of the purification process

Composition of larvae, pupal exuviae and dead adults after each extraction step, as well as composition of the final chitin, were determined and presented in Table 3.

**Table 3:** Composition of insect samples, in terms of minerals, proteins and fibres, before and after each step of the chitin extraction process, in comparison with commercial chitin derived from shrimp shells.

	Raw larvae	DM larvae	DP (unbleached chitin) larvae	Bleached chitin larvae	Commercial chitin
Minerals %	12.5 ± 0.1^a^	2.2 ± 0.01^b^	2.1 ± 0.03^b^	1.4 ± 0.02^b A^	2.2 ± 0.1^A^
Proteins %	38.7 ± 1.6^a^	31.8 ± 2.7^b^	2.5 ± 0.1^c^	1.8 ± 0.02^c A^	1.7 ± 0.3^A^
ADF %	22.0 ± 3.7^c^	35.0 ± 3.6^b^	85.5 ± 2.7^a^	88.5 ± 0.8^a A^	89.2 ± 0.4^A^
ADL %	9.5 ± 2.2^a^	12.9 ± 3.9^a^	8.5 ± 1.6^a^	3.6 ± 0.6^b A^	1.2 ± 0.2^A^
Chitin% (ADF − ADL)	12.4 ± 1.7^d^	22.0 ± 1.9^c^	76.9 ± 4.3^b^	84.0 ± 1.3^a A^	88.1 ± 0.3^A^

	Raw pupal exuviae	DM pupal exuviae	DP (unbleached chitin) pupal exuviae	Bleached chitin pupal exuviae	Commercial chitin
Minerals %	16.0 ± 0.2^a^	2.4 ± 0.2^b^	1.9 ± 0.1^b^	0.5 ± 0.1^c A^	2.2 ± 0.11^A^
Proteins %	30.0 ± 2.8^a^	29.8 ± 2.8^a^	2.3 ± 0.4^b^	2.1 ± 0.4^b A^	1.7 ± 0. 3^A^
ADF %	53.5 ± 1.8^c^	73.6 ± 2.8^b^	90.5 ± 1.5^a^	90.7 ± 0.4^a A^	89.2 ± 0.4^A^
ADL %	28.0 ± 1.2^a^	32.0 ± 1.8^a^	12.7 ± 2.4^b^	3.9 ± 0.1^c A^	1.2 ± 0.2^A^
Chitin% (ADF − ADL)	25.5 ± 0.5^d^	42.9 ± 3.2^c^	77.8 ± 1.7^b^	86.8 ± 0.4^a A^	88.1 ± 0.3^A^

	Raw adults	DM adults	DP (unbleached chitin) adults	Bleached chitin adults	Commercial chitin
Minerals %	8.1 ± 0.5^a^	1.1 ± 0.03^b^	1.27 ± 0.02^b^	0.9 ± 0.1^b A^	2.2 ± 0.1^A^
Proteins %	49.0 ± 0.4^a^	45.6 ± 2.2^a^	1.5 ± 0.5^b^	1.4 ± 0.1^b A^	1.7 ± 0.3^A^
ADF %	23.7 ± 2.4^c^	32.2 ± 1.5^b^	84.8 ± 0.7^a^	86.6 ± 1.2^a A^	89.2 ± 0.4^A^
ADL %	10.8 ± 2.4^a^	11.6 ± 1.1^a^	11.4 ± 0.5^a^	0.8 ± 0.01^b A^	1.2 ± 0.2^A^
Chitin% (ADF − ADL)	12.8 ± 1^d^	20.6 ± 0.4^c^	73.4 ± 0.2^b^	85.3 ± 1.2^a A^	88.1 ± 0.3^A^

Data are expressed as mean ± standard deviation. Different letters in a row indicate significant differences among the different insect samples in the percentage of each component (*p* < 0.05) (data analyzed with one-way ANOVA and Tuckey *post-hoc* test). Same capital letters in a row, indicate no significant differences between chitin extracted from *H. illucens* and the commercial one, for each component (data analyzed with Chi-square test with Yates’ correction).

Minerals were significantly reduced by DM treatment with formic acid for all insect samples (larvae *Χ*^2^ = 6.3, *p* = 0.01; pupal exuviae *Χ*^2^ = 9.5, *p* = 0.002; adults *Χ*^2^ = 4.1, *p* = 0.04), to remain constant during the following steps, with the only exception of pupal exuviae, where bleaching induced a further decrease. It resulted in 82, 85, and 87% DM efficiency for larvae, pupal exuviae, and adults, respectively. Only Hahn et al.^66^ used formic acid for DM of *H. illucens* larval exoskeletons, removing 84% of minerals. A similar efficiency (84.1%) was achieved by Khayrova et al*.*^65^ using hydrochloric acid on *H. illucens* pupal exuviae, while only 47.5% minerals was removed from adult flies applying the same acid. On *H. illucens* prepupae, on the other hand, the use of natural deep eutectic solvents, achieved a DM efficiency of 98%^75^. Our results proved that the use of an organic acid, with less environmental impact and less potentially negative effect on the final chitin^76^, can therefore remove minerals from different *H. illucens* samples with similar or higher efficiency than a mineral acid. This was also demonstrated by other studies on insects and crustaceans: oxalic acid removed a higher percentage of minerals (85%) from house crickets^77^, compared to that obtained using hydrochloric acid, under the same conditions on both flies (39.5%)^78^ and two-spotted crickets (8–21%)^79^, lactic and acetic acid were used for DM on shrimp shells with comparable efficiency to that obtained with hydrochloric acid^76,80^.

Proteins, on the other hand, were decreased significantly by DP treatment with sodium hydroxide in all insect samples (larvae *Χ*^2^ = 37.9, *p* < 0.001; pupal exuviae *Χ*^2^ = 26.2, *p* < 0.001; adults *Χ*^2^ = 57.3, *p* < 0.001) (94, 92 and 97% efficiency), to remain constant until the end of the purification process; in larvae only, a significant reduction of the protein content was already found post DM. It resulted in 94, 92 and 97% DP efficiency for larvae, pupal exuviae, and dead adults, respectively. A slightly lower value (86–87%) was obtained with similar reaction conditions on *Musca domestica* pupae and *Gryllus bimaculatus* adults^78,79^. A higher value (97%) was obtained using natural deep eutectic solvents on *H. illucens* prepupae^75^.

Purity of chitin extracted from the three biomasses of *H. illucens* was expressed as chitin content. The chitin content increased as the extraction process advanced in all insect samples, with the major rise recorded after the DP where unbleached chitin is obtained. The unbleached chitins were very similar to each other (chitin content amounting to 73.4, 76.9 and 77.8%, respectively), except for the residual mineral and protein content, which was lower in adults than in the other ones. The bleaching treatment did not significantly affect the acid detergent fibre (ADF) value and, consequently, the final content of the bleached chitin, amounting to 84, 86.8 and 85.3% for larvae, pupal exuviae and dead adults, respectively. These values highlighted the suitability of the applied purification method for the production of a bleached chitin with a degree of purity similar to the commercially available polymer (88.1%).

Degree of purity of bleached chitin extracted from different insect species mostly ranges from 85 to 97%^64,66,75,81,82^. Given the same insect biomass, the observed differences in chitin purity may be due to the different purification methods applied, in terms of reagents, concentrations, and reaction times, as well as the different methods used to calculate this degree^64,66,75,81,82^.

### Chitosan production

**Figure 2:**
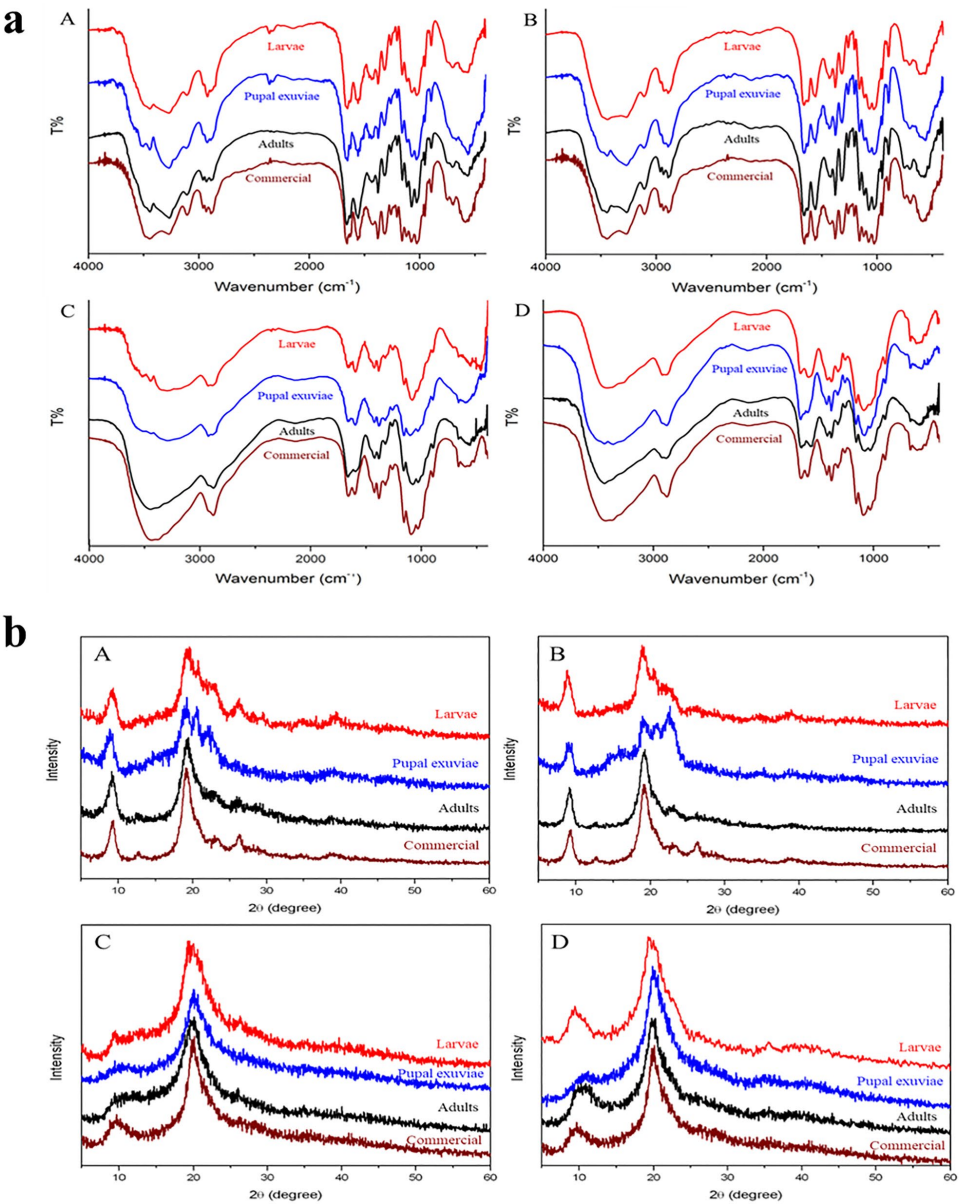
**(a)** FTIR spectra of both unbleached (A,C) and bleached (B,D) chitin (A,B) and chitosan (C,D) samples extracted from *H. illucens* larvae (red line), pupal exuviae (blue line) and dead adults (black line). Commercial chitin and chitosan (wine lines) derived from crustaceans also are reported. **(b)** XRD spectra of both unbleached (A,C) and bleached (B,D) chitin (A,B) and chitosan (C,D) samples extracted from *H. illucens* larvae (red line), pupal exuviae (blue line) and dead adults (black line). Commercial chitin and chitosan (wine lines) derived from crustaceans are also reported.

Chitosan was produced by heterogeneous deacetylation of both unbleached and bleached chitin extracted from the three biomasses of *H. illucens*, thus obtaining six different chitosan samples (Fig. 1b). As expected, chitosan produced from unbleached (Fig. 2b(A–B–C)) chitins were darker than that from bleached chitins (Fig. 2b(D–E–F)), especially adults unbleached chitosan (Fig. 2b(C)). All bleached chitosan also appeared darker than their respective bleached chitins, especially the chitosan of dead adults and pupal exuviae. This browning is probably due to the high temperatures used for the deacetylation reaction, inducing some saccharide dehydration leading to double bonds formation^83,84^.

#### Chitosan recovery and chitosan yield

Yields related to chitin (chitosan recovery after deacetylation) and to the original insect sample were determined for all chitosan samples and are presented in Table 4. For all insect samples, the yield of bleached chitosan related to chitin was higher than the yield of the respective unbleached one (range 25–28%), with the highest values obtained for chitosan from pupal exuviae and adults (42 and 41%, respectively). These significantly different chitosan yield values suggest how, for the same conditions used, the bleaching or not of the chitin influences its deacetylation capacity. Indeed, the unbleached sample presents catechol compounds that, cross-linked to the α-chitin chains, probably hide the acetyl groups and limit the access by NaOH molecules. The reaction parameters should be regulated by trying to force more the deacetylation reaction on the unbleached chitin samples. The yield of chitosan related to the original insect biomass followed the same trend, but with a smaller gap between bleached and unbleached chitosan for each sample. These yield values did not appear to be affected by the bleaching treatment. Yield of chitosan derived from *H. illucens* by heterogeneous deacetylation was reported only by Khayrova et al.^57^ for larvae (53% related to chitin) and Hahn et al.^66^ for larval exoskeletons (47% related to chitin and 16% related to the initial biomass). The yields obtained in the present work are in the range of chitosan produced from insects (2–8%)^32^ and are slightly lower than those of crustacean-derived chitosan (4–15%)^67–69,85,86^. This difference can be explained considering that insect biomass has a higher protein and lipid content than crustaceans, which may lower the final polymer yield^87^. However, as reported for chitin, chitosan yield can be affected by various factors, including the source, the purification methods and the deacetylation treatments applied to chitin^88^.

**Table 4:** Yields (%) related to chitosan, deacetylation degree (DD), viscosity-average molecular weight (M_v_), crystallinity index (CrI %) and crystallite size (nm-D_100_) of chitosan obtained from both bleached and unbleached chitin from *H. illucens* larvae (L), pupal exuviae (PE) and dead adults (A) and a commercial chitosan derived from shrimp shells.

Chitosan sample	Yield (%)					
	Chitosan/chitin	Chitosan/raw	DD (%)	M_v_ (kDa)	CrI (%)	Crystallite size (nm)
L unbleached	25 ± 2.5^c^	3 ± 0.4^bc^	91 ± 0.3^a^	92 ± 0.1^b^	74^a^	3
L bleached	33 ± 0.4^b^	3 ± 0.1^bc^	92 ± 0.7^a^	21 ± 1.4^ h^	77^a^	3
PE unbleached	28 ± 4.5^bc^	8 ± 0.8^a^	83 ± 3.1^b^	55 ± 1.7^e^	78^a^	4
PE bleached	42 ± 1.5^a^	10 ± 0.2^a^	90 ± 4.2^a^	35 ± 4.3f.	80^a^	3
A unbleached	27 ± 2^bc^	2 ± 0.4^c^	91 ± 0.7^a^	62 ± 0.1^d^	79^a^	3
A bleached	41 ± 1^a^	3 ± 0.3^bc^	93 ± 1.4^a^	36 ± 0.5f.	86^a^	3
Commercial	–	–	92 ± 0.7^a^	376 ± 3.3^a^	79^a^	4

Data are expressed as mean ± standard deviation. Different letters in a column indicate significant differences (*p* < 0.05) in the yields, DD, M_v_ or CrI among the samples (data analyzed with Chi-square test with Yates’ correction).

### Chitin and chitosan characterization

#### Fourier-transformed infrared spectroscopy (FTIR)

Spectra resulting from FTIR analysis of both unbleached and bleached chitins from *H.* *illucens* larvae, pupal exuviae and dead adults are shown in Fig. 2a(A,B), in comparison to the commercial crustacean-derived chitin. All the characteristic peaks of chitin were detected in all samples at their specific wavelengths: 1310–1320 cm^−1^ (CN-stretching, amide III), 1550–1560 cm^−1^ (NH-bending, amide II), 1650–1655 cm^−1^ (CO-stretching, amide I), 3100–3110 cm^−1^ (NH-symmetric stretching), 3255–3270 cm^−1^ (NH-asymmetric stretching) and 3430–3450 cm^−1^ (OH-stretching). The α-form was confirmed for all chitin samples produced from *H. illucens* by observing the two Amide I band splits, around 1620 and 1650 cm^−1^^56,70,89–91^. The spectra of all chitins showed a structural similarity with the commercial polymer. No significant differences were observed either among chitin extracted from the different starting insect materials, in accordance to other authors^63,72,73^, or for each sample, between the bleached chitin and the respective unbleached one. Some differences in peak wavelengths are probably due to the different natural sources and the extraction process applied. Chitin acetylation degree (AD) was also determined from the spectra (Table 5).

**Table 5:** Acetylation degree (AD %), crystallinity index (CrI) and crystallite size (nm) resulted from FTIR and XRD analysis of both bleached and unbleached chitin samples extracted from *H. illucens* larvae (L), pupal exuviae (PE) and dead adults (A) and a commercial chitin derived from crustaceans.

Chitin sample	AD (%)	CrI (%)	Crystallite size (nm)
L unbleached	92^ab^	90^b^	4
L bleached	94^ab^	84^b^	5
PE unbleached	91^ab^	67^c^	4
PE bleached	89^b^	62^c^	5
A unbleached	98^a^	96^ab^	5
A bleached	96^ab^	93^ab^	6
Commercial	91^b^	98^a^	6

Different letters in a column indicate significant differences (*p* < 0.05) in the AD or CrI among chitin samples (data analyzed with Chi-square test with Yates’ correction).

The AD values of all chitin samples are within the range of AD reported for both insect-derived chitin and the commercial one (80 -100%), considering only the AD determined by FTIR^63,89,92–94^. Results of the present work were also similar to those already reported for chitin derived from *H. illucens*^56,63,64^. The AD of chitin obtained from adults was the highest. These comparable values among the samples (larvae, pupal exuviae, dead adults) of *H. illucens* indicated that AD was not subject to large variations in the growth stages, confirming that reported by other authors for the same insect^63^ and other species^82^.

Spectra resulting from FTIR analysis of chitosan samples are shown in Fig. 2a(C,D), in comparison with the commercial one. As reported for chitin, characteristic peaks confirming the identity of chitosan were detected, specifically the NH-bending (amide II) and CO-stretching (amide I) bands around 1655 (amide I) and 1590 cm^−1^ (NH_2_ bending), respectively^86,90,91,95,96^. No significant differences were observed between the spectra of bleached chitosan and the respective unbleached sample for adults only. The N–H and O–H stretching bands in the 3000–3600 cm^−1^ region are more complex for chitin than for chitosan, in agreement with the presence of different groups (amine or amidic N–H, for instance). Moreover, this region is affected by inter-macromolecular interactions that seem to be more complex in chitin. These interactions are connected to supramolecular organization (presence of hydrogen bonds, formation of crystals, etc.), depending on material morphology. The deacetylation, being a chemical reaction that affects the chitin morphological structure, had reasonably affected intermolecular and supramolecular organization, as deductible by comparing chitin and chitosan spectra.

#### X-ray diffractometry (XRD)

XRD analysis was performed to determine crystallinity of chitin and chitosan from *H. illucens*.

The spectra obtained for chitins are shown in Fig. 2b(A,B). Similarly to the commercial sample, all chitins showed the significant sharp peaks at 9° and 19° and the three/four weak peaks around 13°, 21°, 23° and 26°, confirming the α-form of the polymer^70,89,97^. No significant differences were found in the spectra between unbleached and bleached chitin; only both chitins derived from pupal exuviae were different for the presence of more intense peaks between 19° and 26° (Fig. 2b(A,B). The peaks of all chitin samples were found to be very similar to those reported for other insect species, in range 9°–26°^89,92,98,99^ and for *H. illucens* itself^56,64,72,73,97^.

The determination of the CrI from the XRD data revealed significant differences among chitins derived from various developmental stages, comparing them to commercial chitin (Table 5). Generally, all bleached chitins had a slightly lower CrI than the respective unbleached samples (although not significantly). This suggests a possible detrimental effect of the bleaching treatment on the crystalline structure of polymer.

Adults-derived chitin was the most crystalline and not different from commercial one, followed by larvae with slightly lower values, while the lowest CrI were obtained from pupal exuviae derived chitins with values below 70%. Crystallite size was similar among all chitins, including the commercial one (Table 5).

Similar results were also reported by other authors for chitin produced from different biomasses of *H. illucens*, with adult chitin always being more crystalline than chitin from other stages^56,72,73^. It was inferred that the crystallinity of chitin increases gradually, thus showing a more ordered structure, at the life stages of the dipteran, particularly from pupa to adult^56,72^.

The CrI values of crustacean and insect chitin fall within a wide range, from 40 to 90%, mainly 60–80%, as they are depending on the source, in terms of species, growth stage and gender, and on the purification process^32,74,75,96,97,99–101^. According to its crystallinity, chitin can be used in different fields: a more amorphous chitin (low CrI) has absorbent properties that make it effective in removing contaminants, such as heavy metals, and therefore useful in water treatment and industrial applications^102^; on the other hand, the high crystallinity of chitin can be a positive aspect for the formulation of chitin nanofibrils, applied in the cosmetic and biomedical field^103,104^.

XRD patterns of both unbleached and bleached chitosan are shown in Fig. 2b(C,D).

In the XRD analysis of all chitosan samples, the two main sharp peaks around 10° and 20°, were observed. These peaks were similar to those reported for insect-based and crustacean chitosan^32,86,89,105,106^. As reported for chitin, no significant differences were found in the spectra between unbleached and bleached chitosan; bleached chitosan derived from larvae and pupal exuviae were different from their respective unbleached samples by the presence of a more and less intense peak at 9°, respectively. CrI values of chitosan samples were all statistically similar, including the commercial one, and ranged from 74 to 86% (Table 4). There is a tendency for bleached samples to be slightly more crystalline than unbleached ones, although not significantly. Due to the lack of studies on the effect of chitin bleaching on the crystallinity of chitosan, it was not possible to compare results of the present work with others in the literature. The crystallinity of chitosan obtained from *H. illucens* was higher than that reported for the other insects (33–69%)^86,105–108^ and more similar to that of commercial one. Crystallite size was similar among all chitosan samples, including commercial one (Table 4). The crystal dimension decreased due to deacetylation because of the chemical treatment. Interestingly, crystallinity decreased in chitosan with respect to larval and adult chitin, but increased in pupal exuviae chitosan, suggesting that in pupal exuviae samples recrystallization can more extensively occur. Indeed, the alkaline treatment with 12 M NaOH and the successive steps, lead to a solubilization of chitosan in acetic acid solution. Hence, the crystallinity is generated after the final reprecipitation of the polymer. In the case of chitosan from adult chitin a higher order in the macromolecular chain is thus present, allowing a more extensive organization in crystals.

#### SEM

**Figure 3:**
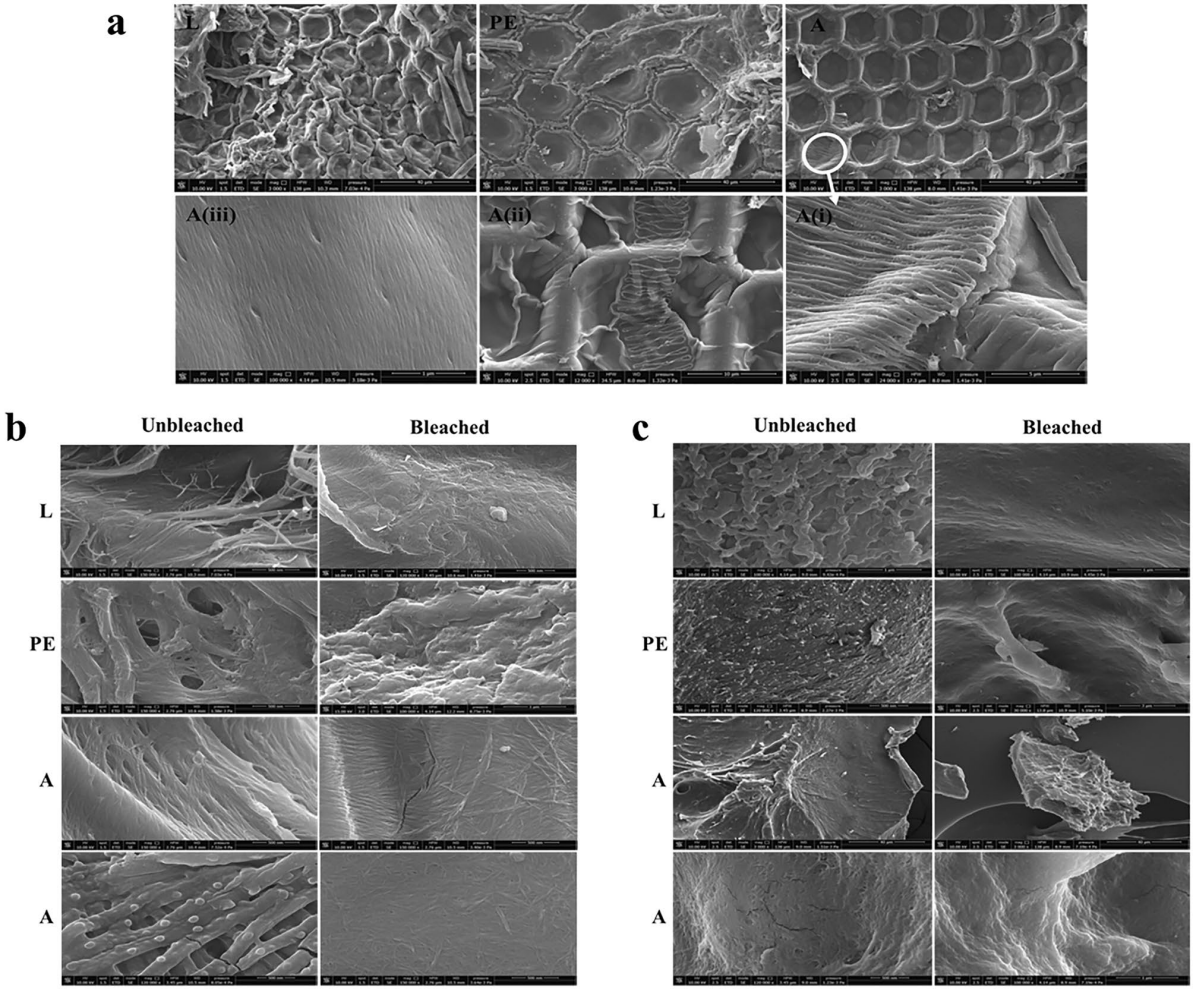
SEM images of **(a)** honeycomb-like structure of chitin extracted from *H. illucens* larvae (L), pupal exuviae (PE), with focus on adult chitin (A, Ai, Aii, Aiii). Bars in (L, PE, A):40 µm; bars in (Ai-iii): 5, 10 and 1 µm, respectively **(b)** Chitin from *H. illucens* larvae (L), pupal exuviae (PE) and dead adults (A) before (unbleached) and after (bleached) bleaching treatment Bars (L, PE, A): 1 and 500 µm. **(c)** Chitosan from *H. illucens* larvae (L), pupal exuviae (PE) and dead adults (A) before (unbleached) and after (bleached) bleaching treatment. Bars in (L): 1 µm; bars in (PE): 500 and 3 µm; bars in (A): 1, 40 and 500 µm.

The surface morphologies of chitin and chitosan produced from *H. illucens* were observed by SEM and shown in Fig. 3a–c. First, chitin in the different sources, at 3000 × magnification, exhibited a structure with honeycomb-like arrangement, based on the repetition of square, pentagonal, and hexagonal units (Fig. 3a). Looking closer (magnifications 12,000–150,000×), the chitin samples showed significant surface differences (Fig. 3a,b). Various studies reported for chitin four different surface morphologies, such as (1) rough and dense surface without nano/microfibers and pores, (2) surface with combination of nano/microfibers and pores (the most common morphology), only fibrillar (3) and porous (4) surface^109,110^. All unbleached and bleached chitin samples (Fig. 3b) consisted of scattered nanofibers with a diameter of about 30–50 nm. The surface complexity was found the highest for the adult chitin, whereas it becomes lower for pupal exuviae chitin and finally for larval chitin. Bleaching treatment had not significantly affected the chitin morphology of larvae and pupae exuviae whereas, for adult chitin, this process had removed some round particles from the fibrous arrangement. Chitins from larvae had a rough surface with broken fibers and an absence of pores that were present in chitins derived from exuviae and from adults. The surface of pupal exuviae chitins were denser than those of larvae, and not much porous. The micrometric morphology is evidently less regular than the one of adult chitin. Nanometric and micrometric holes peculiar to adult chitins revealed the presence of oriented nanofibers delimiting them (Fig. 3a(Ai–iii)). As reported by Purkayastha and Sarkar^56^ and Soetemans et al*.*^64^, the chitin derived from *H. illucens* adults showed different structural morphologies explained by the various extremities constituting the fly's body. In agreement with the literature, the morphology of insect chitin can vary not only depending on the different species, and growth stage, but also on the genus and body part^89,90,97,109^. Indeed, Kaya et al*.*^82,90,109^ had described many different morphologies for adults of *V. crabro*, for a honeybee and between the females and males of some grasshopper species. Our results confirmed the presence of different chitin arrangement among different biomasses of *H. illucens*^56,64,72,73,97^. According to its morphology, chitin can be used for different applications; particularly, chitin with a fibrillary surface is suitable for textile industry, while with a porous structure it can be used in tissue engineering and drug delivery^3,89^.

All chitosan samples obtained from the larvae, pupal exuviae and adults of *H. illucens* (Fig. 3c) showed a rough but less fibrillated structure when compared to the respective chitin ones, demonstrating that deacetylation step altered the chitin structure, making it more homogeneous and less fibrillated. Indeed, the chemical chitin deacetylation deeply impacted its morphology. Chitosan nanofibers can be formed after reprecipitation of the solid polymer from acidic solution or being generated by modifying the previously existing chitin ones. Due to the higher morphologic complexity of adult chitin, we can hypothesize that the latter mechanism dominates in this case. On the contrary, the other samples, being less complex, can be more deeply modified resulting in a more effective formation of new nanofibrils. However, this fibrillated structure was more evident in unbleached than bleached samples, suggesting an influence of bleaching on the capacity of deacetylation to occur. As for chitin, the chitosan samples also had some pores on their surface. Due to the lack of studies on the surface morphology of chitosan produced from *H. illucens*, it was not possible to compare results of the present work with others in the literature. In an attempt to correlate the final physical–chemical properties of chitosan to the starting chitin structure, it is evident that the biomass of *H. illucens* is relevant. Adult chitin, showed a very high starting crystallinity and AD (linked to the high regularity of macromolecular structure), an intermediate value of viscosity average molecular weight (M_v_) and a complex morphological structure resulted in a final chitosan showing the highest values of deacetylation degree (DD) and crystallinity. Hence, the morphological complexity of the sample is not significantly affected by these parameters. Pupal exuviae, showing the lowest crystallinity (linked to the lowest AD), an intermediate morphological complexity and a low M_v_ resulted in the lowest DD and intermediate crystallinity.

Finally, larval chitin, showing intermediate values of crystallinity, AD, the lowest morphologic complexity and the highest M_v_, resulted in a chitosan with an intermediate DD and the lowest value of crystallinity. A low M_v_ in chitosan can be thus considered important for having a final high crystallinity, in agreement with the observations of Osorio-Madrazo et al.^111^. A high starting AD seems another important element for having a high DD and crystallinity in the final chitosan. The bleaching, resulting in a strong decrease in M_v_, determines an increase in crystallinity of the final chitosan. In general, despite different morphological structures were evidenced for the different biomasses, they did not extensively affect these important chitosan features, suggesting that the chemical attack due to deacetylation strongly modified the starting chitin morphological structure.

#### Potentiometric titration of chitosan

The DD of all chitosan samples produced from *H. illucens* is reported in Table 4. DD of all chitosan samples was around 90%, similarly to that of commercial chitosan, except for the unbleached sample from pupal exuviae (83%). DD reported by other authors for the chitosan produced from *H. illucens* was similar^64^ or lower^57,66^ than that measured in the present work, ranging from 40 to 90%. In most cases, chitosan from insects has a DD between 62 and 98%^32^, in accordance with the average DD reported for crustacean-derived chitosan (56–98%)^112^. DD is an important parameter that influences different properties of chitosan, and it is dependent on the deacetylation conditions applied, in terms of temperature, reaction time and NaOH concentration; generally, higher temperatures can increase the DD^113^. A high DD enhances the antimicrobial activity of chitosan against certain bacterial species^114,115^.

#### Viscosity-average molecular weight (M_v_)

Molecular weight (Mw) for all chitosan samples was determined via viscometry and is reported in Table 4. M_v_ of all chitosan samples produced from *H. illucens* was much lower (from 21 to 92 kDa) than that of commercial chitosan (376 kDa). The M_v_ of chitosan samples from unbleached chitin was always higher than that of the respective bleached samples. These results confirm an effect of the bleaching treatment on the viscosity and M_v_ of the final chitosan^113,116^. In particular, the bleaching reasonably decreased the Mw of the starting chitin inducing some scissions of polysaccharidic chains; probably the absence of catechol compounds making the polymer chains more accessible to partial hydrolysis too. Previous studies reported a M_v_ for insect chitosan in the range of 426–450 kDa^107,117^. Considering the Mw determined by size exclusion chromatography techniques, insect chitosan ranges from 26 to 300 kDa^32^, whereas chitosan from crustaceans is reported to range from 100 to 1000 kDa^69,118^. Much lower values of both M_v_ and Mw, between 3 and 10 kDa, have also been reported for insect chitosan, which are more similar to those obtained in the present work^89,95,119^. Extremely low Mw can be due to a polysaccharide depolymerization caused by strong deacetylation conditions in terms of incubation time and temperature^32,69^. Indeed, the same severe conditions can reduce the viscosity and thus M_v_ of the chitosan samples. We noticed that a lower M_v_ is generally linked to a higher crystallinity. Indeed, bleached chitosan samples had a lower M_v_ and higher CrI values compared to the respective unbleached samples. Mw can greatly affect physicochemical properties and biological activity of chitosan, often controlled by the macromolecular dimension. It is generally reported that chitosan with low Mw (< 150 kDa) has better antibacterial properties than high-Mw chitosan, since it can easier cross the cell wall of bacteria^120,121^.

#### Chitosan film formation ability

All chitosan samples produced from *H. illucens* larvae, pupal exuviae and dead adults were able to form uniform homogeneous films. Figure 1c provides the photographic documentation. On the optical properties point of view, film of chitosan from adults (Fig. 1c(C,F) were the most different from all other samples, including commercial one: the unbleached chitosan film retained its dark brown colour, whereas the bleached one was much lighter but still more pigmented than the other bleached chitosan films.

## Conclusion

Recently, number of farms of bioconverter insects, such as *H. illucens*, used for industrial protein feed production and organic waste management, is increasing. This process acquires greater economic value through the exploitation of the side streams resulting from the insect processing; indeed, the latter, having no other destination than the disposal as waste, could be inserted into a new productive cycle for the purification and production of valuable macromolecules, such as chitin and chitosan, which can then be functionalized and put back on the market. This study is the first comprehensive report on the isolation, production and characterization of chitin and chitosan derived from three different biomasses of *H. illucens*: larvae, pupal exuviae and dead adults. The latter two are among the main waste products, readily available and easy to collect from insect breeding facilities. Currently, the production of chitin and chitosan from insects is carried out on a laboratory scale, using the same procedures as for crustaceans and lacking numerical data providing a quantitative assessment of the effectiveness of chitin extraction processes. The method applied in this work was effective in significantly reducing the percentage of minerals and proteins contained in the insect raw samples, resulting in a chitin of similar purity to the commercially available one. Pupal exuviae, as expected, were the biomass richest in chitin, with the highest yield of both chitin and chitosan, thus representing the biomass of choice for the polymers production. FT-IR and XRD spectra of all chitin and chitosan samples also confirm their similarity to commercial ones, thus validating *H. illucens* as an alternative source of these biopolymers. The starting biomass of *H. illucens* (larvae, pupal exuviae and dead adults) provided the main driver for modulating the morphology of chitins, as well as the physical–chemical properties of final chitosan products.

Starting from this study, it will then be possible to proceed by relating the specific chemical-physical and morphological characteristics of the polymers with the applications of interest. In the case of our chitin samples, due to their high degree of crystallinity, they represent ideal candidates for the chitin nanofibrils formulation, applicable in the cosmetic and biomedical fields, while their fibrillar structure makes them suitable for producing fibrous manufacts.

From the characterization of chitosan produced from *H. illucens*, the high versatility of the polymer, suitable for different applications, is evident. Indeed, the optimal characteristics of this polymer change according to its use, making it difficult to define univocal optimal parameters for its production. Its filmogenic capacity makes it suitable to be used as preservative coating in the food industry; the low M_v_, associated with a low viscosity and a high DD, could instead be encouraging features for antimicrobial activity and for future biomedical and pharmaceutical applications. The results obtained from this work are encouraging and represent a starting point for further investigations oriented to the optimization of the current chemical purification and characterization processes, to the development of "green" extraction processes, as well as on the specific applications of the final polymer.

## Materials and methods

### Sample collection and preparation

*H. illucens* larvae, pupal exuviae and dead adults were provided by Xflies s.r.l (Potenza, Italy). Larvae were reared on a standard Gainesville diet (30% alfalfa, 50% wheat bran, 20% corn meal)^122^ and were collected in the last larval stage. Pupal exuviae were taken after the adults emerged, and dead adults were sampled from the flight cages at the end of their life cycle. All samples were washed with distilled water, dried in an oven at 60 °C for 24–48 h and then ground into powder with a lab grinder (Waring Commercial Stamford, USA). Commercial chitin and chitosan, derived from crustacean shells, were purchased from MP Biomedicals (Irvine, California, USA) and Sigma-Aldrich (St. Louis, Missouri, USA), respectively.

### Determination of proximate composition of raw samples

Raw larvae, pupal exuviae and dead adults were analyzed in order to determine their composition in terms of minerals, proteins, fats and fibers. The results of the composition determination were related to the dry mass, which was also calculated. Dry mass of all insect samples was determined by drying in an oven (Conlabo s.r.l., Potenza, Italy) overnight at 100 °C, according to the standard method EN ISO 11465:1993. The mineral content was determined after incineration of the insect samples at 550 °C in a muffle furnace (Gefran 1001, Provaglio d'Iseo, Italy), according to the standard method EN ISO 14780:2017. The protein content was estimated by spectrophotometric method^123^, after incubation of the samples in a protein lysis buffer prepared with 5% sodium dodecyl sulphate (Sigma-Aldrich St. Louis, Missouri, USA), 50 mM acetic acid (Sigma-Aldrich St. Louis, Missouri, USA), 10 mM boric acid (Sigma-Aldrich St. Louis, Missouri, USA), 4 M urea (Sigma-Aldrich St. Louis, Missouri, USA) and 10% glycerol (Sigma-Aldrich St. Louis, Missouri, USA). Samples were measured with a Nanodrop ND1000 spectrophotometer at a wavelength of 280 nm (A280). Due to the ratio 260/280, a wavelength of 260 nm (A260), indicative of contamination of nucleic acids and catecholamines, was also determined. The protein concentration was thus determined according to the Eq. (1) by Layne^124^ and related to the weight of the sample:

1 $${{Protein}} \, {(\%)}\, {=}\frac{\left({1.55\, \times \,A280}\right){- }\left({0.76\, \times \,A260}\right)}{{weight \,of \,sample } \, \text{(g)}} \, {\times }\,{100}$$

Lipid content of the raw insect samples was determined by Soxhlet (Sigma-Aldrich, St. Louis, Missouri, USA) extraction using *n*-hexane (Sigma-Aldrich St. Louis, Missouri, USA).

Although the cellulose is fully absent in insects, the structural similarity between chitin and cellulose was exploited to determine the chitin content. The procedure consisted of two steps after which different fibre components, acid detergent fibre (ADF) and acid detergent lignin (ADL), were obtained, according to the method used by Hahn et al*.*^59^. Considering that ADF provided the fibre content, in terms of chitin and catecholamines, and ADL only the one of catecholic compounds, chitin value was calculated using the Eq. (2):

2 $${Chitin } \, {(\%)} { = ADF } {(\%)}{ - ADL } {(\%)}$$

### Extraction of chitin

The extraction of chitin from insect samples (larvae, pupal exuviae and dead adults) was carried out based on the process reported by Hahn et al*.*^66^.

#### Demineralization (DM)

To remove minerals, mainly calcium carbonate, the powdered samples were suspended in 0.5 M formic acid (Sigma-Aldrich St. Louis, Missouri, USA) (solid: liquid ratio 1:10 (m/v)) and stirred for 1 h at room temperature. The demineralized material was then filtered through a sieve cloth and washed to neutral with distilled water. The washed samples were dried at 60 °C overnight in oven.

#### Deproteinization (DP)

Proteins were removed from demineralized samples by treatment with 2 M sodium hydroxide (Sigma-Aldrich St. Louis, Missouri, USA) (NaOH) (solid:liquid ratio 1:10 (m/v)), stirring for 2 h at 80 °C. The deproteinized material was again filtered through a sieve cloth, washed to neutral with distilled water and dried at 60 °C overnight in oven. After DP unbleached chitin was obtained.

#### Bleaching

Unbleached chitin was treated with a solution of 5% (v/v) hydrogen peroxide (H_2_O_2_) (Sigma-Aldrich St. Louis, Missouri, USA) (solid: liquid ratio 1:20–30) for 30–60 min at 90 °C, under stirring, according to Hahn et al.^125^. The bleached samples were filtered using filter paper, washed to neutral pH with distilled water and finally dried at 60 °C overnight in oven. After this treatment, bleached chitin was obtained.

### Assessment of the chitin purification process

Samples were analyzed after each step of the chitin purification process to determine changes in their composition, in terms of minerals, proteins, fibers and chitin content, according to methods described in “Determination of proximate composition of raw samples” for raw insect samples.

The efficiency of DM and DP was measured by comparing the mineral and protein content of the samples before and after the respective treatment, according to the following Eqs. (3, 4):

3 $${DM \, efficiency }\, {(\%)} { = } \frac{{{mineral}} \, {(\%)} \,{ raw \, sample - mineral} \, { (\%)} \, { DM\, sample }}{{mineral\, (\%) \,raw \,sample}} \,{ \times } \,\text{ 100}$$

4 $${DP \, efficiency } \, {(\%)}{ = }\frac{{{protein}} \, { (\%) }{raw \, sample - protein} \, { (\%)} \, { DP \,sample }}{{protein } \, {(\%)} \, { raw \, sample}} \, \times { 100}$$

The yield of both unbleached and bleached chitin was also calculated as a ratio between the chitin dry weight and that of the initial insect sample (Eq. 5):

5 $${Chitin\, yield } \, {(}{\%)}\, { = }\, \frac{{dry\, weight\, of\, chitin}\text{ (g)}}{{dry\, weight\, of\, the\, original\, raw \,insect\, sample} \text{ (g)}} \, \times {100}$$

### Chitosan production

Chitosan was obtained by heterogeneous deacetylation of both unbleached and bleached chitin extracted from the three *H. illucens* biomasses (larvae, pupal exuviae and dead adults). Chitin samples were suspended in 12 M NaOH (Sigma-Aldrich St. Louis, Missouri, USA) (solid: liquid ratio 1:20) and stirred for 4 h at 100 °C. At the end of the reaction, the suspension was filtered using filter paper and the solid residue was washed to neutrality with distilled water. After washing, the deacetylated material was incubated in 1% (v/v) acetic acid (Sigma-Aldrich St. Louis, Missouri, USA) at room temperature for 48 h, under stirring. The mixture was then centrifuged at 10,000 rpm for 5 min and the supernatant was collected. The solution was adjusted with 6 M NaOH (Sigma-Aldrich St. Louis, Missouri, USA) to a pH 8 and incubated overnight at 4 °C, in order to precipitate the solubilized chitosan. The suspension was centrifuged again, so the chitosan was collected and washed with distilled water, to remove the remaining acetate adsorbed by chitosan^66^. The final product was freeze-dried and stored at room temperature.

The yield of both chitosan, unbleached and bleached, was firstly calculated for all the samples, similarly to chitin, according to the following Eq. (6):

6 $${{Chit}}{osan\, yield } \, {(\%)} \,{ = } \, \frac{{dry\, weight\,} {{of}} \, {{chitosan}}\text{ (g)}}{{dry\, weight\, of\, the\, original\, raw\, insect\, sample\, }\text{(g)}} \, \times {100}$$

### Chitin and chitosan characterization

All chitin and chitosan samples were analysed in order to characterize them and assess their quality and suitability for potential applications. Fourier-transform infrared spectroscopy (FTIR), X-ray diffraction (XRD) analysis, and scanning electron microscopy (SEM) were performed on both chitin and chitosan. Additionally, the deacetylation degree (DD), intrinsic viscosity, viscosity-average molecular weight (M_v_), and film forming ability of chitosan were determined.

#### Fourier-transformed infrared spectroscopy (FTIR)

The IR transmission spectra of the chitin and chitosan samples were recorded using a Jasco 460Plus IR spectrometer. The samples were scanned with a resolution of 4 cm^−1^ and 100 accumulations and the transmittance values (T%) were evaluated in the range of wavelength 4000–400 cm^−1^. The resulting spectra were processed using JASCO Spectra Manager software. For analysis, chitin and chitosan dry and pulverised samples were mixed with KBr (potassium bromide) and the mixture was pressed to obtain tablets with a diameter of 1 cm. The AD of chitin samples, attributed to the C=O stretching of amide group, was estimated by evaluating the ratio between the area of the bands centred respectively at 1660 and 2700 cm^−1^, according to the Eq. (7) by Weißpflog et al*.*^126^:

7 $${{AD}} \, {(\%)}\,{ = }\,\frac{\frac{{\text{A}}{1660}}{{\text{A}}{2700}}\,{ \times100}}{1.33}$$

#### XRD

The X-ray diffraction spectra of the chitin and chitosan samples were measured using an X-ray diffractometer (X’Pert PRO, Philips) with Cu Kα irradiation (40 kV, 32 mA) and 2θ with a scan angle between 5º and 50º at a scan speed of 0.04º s^−1^. The crystallinity indexes(CrI) of chitin and chitosan were calculated according to the Segal method^127^ (Eq. 8):

8 $${{CrI}} \, { (\%) =} \frac{{Ic- Ia}}{{Ic}} \, \times \, { 100}$$

where *Ic* is the intensity of the highest diffraction peak (crystalline portion) and *Ia* is the minimum intensity between major peaks (amorphous band)^127^.

The size of the crystallites of each chitin and chitosan sample was determined as well, using the Scherrer Equation^128^ (Eq. 9):

9 $$D \, \left( {nm} \right) = k\lambda /\beta cos\theta$$

where D is the size of the crystallites (nm), k = 0.9, λ is the wavelength, β is the width at half height of the peak analysed, while θ is the corresponding diffraction angle.

#### SEM

The surface morphologies of the chitin and chitosan samples were examined by analyzing the powder samples by using a field emission FEI Quanta 450 FEG electron microscope.

#### Determination of chitosan DD

The DD of all chitosan samples was determined by potentiometric titration, according to the method of Jiang et al.^129^, that exploits the pH sensitivity of the amino groups of the polymer chain, which are protonated under acidic conditions. To confirm the validity of the method used for DD determination, a commercial chitosan (Sigma-Aldrich) with a known DD was used as a reference.

#### Determination of chitosan M_v_

The M_v_ of all the chitosan samples was determined by measuring the intrinsic viscosity of the respective chitosan solutions. Intrinsic viscosity (*η*) of chitosan was determined using an Ostwald capillary type viscometer (Fisher Scientific, Waltham, Massachusetts, USA), according to the method by Singh et al.^130^. The M_v_ of each chitosan sample was then calculated using the following Mark–Houwink–Sakurada equation (10)^131^:

10 $$\left[{\eta}\right]= \text{K} {\text{M}}_{\text{v}}^{-\alpha}$$

where [η] is the intrinsic viscosity, and K and α values were determined by Sing et al*.*^130^.

#### Film forming ability

The ability to form films is a characteristic property of chitosan that is particularly important for its application as a coating agent. It was evaluated for each chitosan produced according to the method reported by Hahn et al.^66^. Briefly 0.1 g chitosan was dissolved in 10 ml of 1% (v/v) acetic acid and the solution was poured into a 100 mm diameter polystyrene Petri dish. The solutions were left to dry at room temperature for 3 days, leaving the lids of the Petri dishes open. The dried chitosan films were photographically documented, visually evaluating their homogeneity and transparency.

### Statistical analysis

All measurements were performed in triplicate and the data were expressed as average ± standard deviation. The data distribution was first verified using the Shapiro-Wilk test, in order to choose appropriate statistical tests to detect significant differences (p < 0.05). Normally distributed data were analyzed with the one-way Anova with Tukey’s post hoc test. Data with non-normal distribution were analyzed with a non-parametric test (the Mann–Whitney U test). Pairwise comparisons of percentage data were performed with the Chi-square test with Yates’ correction. For pairwise comparisons of non-percentage data, the t-test with Welch’s correction was used. All statistical analyses were performed using GraphPad Prism version 6.0.0 for Windows (GraphPad Software, San Diego, California USA) and JMP, Version 7 (SAS Institute Inc., Cary, NC, 1989–2021). All measurements of FTIR, XRD and SEM were done in triplicate and, after confirming similarity, one of each sample was shown.

## Data Availability

The datasets used and/or analysed during the current study are available at the following link: https://drive.google.com/drive/folders/1rzsSX6DFYBiBDDnq5c0vpOhXbMKZ1GgW?usp=sharing.
